# Subjective Memory Complaints and Decision Making in Young and Older Adults: An Event-Related Potential Study

**DOI:** 10.3389/fnagi.2021.695275

**Published:** 2021-11-03

**Authors:** Ruth Garrido-Chaves, Vanesa Perez, Mario Perez-Alarcón, Isabel Crespo-Sanmiguel, Tiago O. Paiva, Vanesa Hidalgo, Matias M. Pulopulos, Alicia Salvador

**Affiliations:** ^1^Laboratory of Social Cognitive Neuroscience, Department of Psychobiology, IDOCAL, University of Valencia, Valencia, Spain; ^2^Laboratory of Neuropsychophysiology, Faculty of Psychology and Education Sciences, University of Porto, Porto, Portugal; ^3^Department of Medical Imaging, University of Porto, Porto, Portugal; ^4^Department of Psychology and Sociology, Area of Psychobiology, University of Zaragoza, Teruel, Spain

**Keywords:** subjective memory complaints, aging, decision making, Iowa gambling task, FRN, P3

## Abstract

Subjective memory complaints (SMCs) may affect decision-making processes. This study aimed to investigate the neuronal correlates of feedback processing during a decision-making task in young and older adults with and without SMCs. Event-related potentials and behavioral performance during the Iowa gambling task were recorded in a total of 136 participants (65 young adults, 71 older adults). The participants were divided into two groups according to their SMCs (with SMCs: *n* = 60, without SMCs: *n* = 76). Feedback-related negativity (FRN) and P3 were analyzed in the feedback stage of the decision-making process. Older adults with SMCs scored worse in the ambiguity phase than older adults without SMCs. The FRN latency was longer for losses in older people with SMCs than in older people without SMCs in the first block. No significant differences between young and older adults with and without SMCs were observed in the other ERP measures. Compared to young adults, older adults showed delayed latency in the FRN component and reduced amplitudes and delayed latency in the P3 component. In conclusion, older people with SMCs present deficits in the decision-making process. These deficits are observed at the behavioral level, but also in neural mechanisms of early feedback processing of negative outcomes.

## Introduction

Subjective memory complaints (SMCs) represent an individual’s perception of subtle changes in memory in the absence of an objective memory impairment (Steinberg et al., 2013). Several studies have reported that older adults with SMCs are more vulnerable to developing future cognitive decline that can lead to mild cognitive impairment (MCI) and dementia than older adults without SMCs (Waldorff et al., 2012; Jessen et al., 2014; Mitchell et al., 2014; Mendonça et al., 2015; Peters et al., 2019). Although the main reasons for the appearance of SMCs are still being debated (Peavy et al., 2013; Ruiz-Sánchez de León and Pedrero-Pérez, 2013; Carrasco et al., 2017; Molina-Rodríguez et al., 2018), some authors have proposed that poorer executive function (EF) performance is one of the most common characteristics of this population (e.g., Ruiz-Sánchez de León et al., 2010; Steinberg et al., 2013; Oh et al., 2017). Given that EF are necessary prefrontal cortex-dependent mental activities to effectively adapt to the environment and achieve goals (Kaller et al., 2010; Tsuchida et al., 2010; Barbey et al., 2013; Diamond, 2013), investigating the EF performance of individuals with SMCs may offer critical information to better understand how SMC-related deficits may affect daily life activities.

Decision making is considered a complex EF process related to the activity of the prefrontal cortex (Bechara et al., 2000; Diamond, 2013; Hebscher and Gilboa, 2016). It involves making a choice between various alternatives depending on the results and the significance of all the options (Bechara, 2005). The Iowa gambling task (IGT) is one of the most widely used assessment tools to simulate real-life decision making (Bechara et al., 1994). During the task, participants have to win as much money as possible by freely choosing one deck from four that can lead to a loss or a win. There are two advantageous decks and two disadvantageous decks that yield higher benefits or higher long-term losses, respectively. Participants receive feedback after each choice, and they tend to adapt their choices in the following trials based on their processing of the feedback. The IGT has two differentiated phases: (1) the ambiguity phase (40–50 first trials), when participants do not yet estimate the benefits of the decks, and (2) the risky phase (60–50 later trials), when participants are able to estimate the contingencies of the decks. Normally, healthy participants learn the rule after 40–50 trials, continuing to choose advantageous decks, whereas patients with orbitofrontal cortex dysfunction cannot learn the contingencies of the decks and perform poorly on the IGT because they continue to choose disadvantageous decks (Bechara, 2000; Struglia et al., 2011). Moreover, recent discoveries in neuroimaging indicate that task performance is associated with the activation of multiple brain areas of the frontal cortex, including the ventromedial and dorsolateral areas of the prefrontal cortex (Ouerchefani et al., 2017). Furthermore, in the case of SMCs, Smart and Krawitz (2015) observed difficulties in the risky phase of the IGT in older people with SMCs. Although no group differences in the overall performance were found, the authors observed that, compared to the control group, people with SMCs emphasized the current result more than past results. A possible explanation for these results would be that the two groups differ in the way they process the feedback received after each choice. Using the functional magnetic resonance imaging (fMRI) technique, a recent study by Hu et al. (2017) observed a decrease in future-oriented decision making in participants with SMCs when compared to a control group of older adults. They observed that future imagination increased future-oriented choices and was associated with increased activation in the medial frontal polar cortex, right insular cortex, and anterior cingulate cortex in controls, but not in SMC individuals. Together, these two studies provide important evidence suggesting that SMCs would potentially influence the decision-making process, and that individuals with and without SMCs may differ in prefrontal cortex activity when engaging in decision-making tasks. However, little is known about the neurophysiological mechanisms underlying these differences, or whether there are differences in the time course of the feedback processing.

Event-related potentials (ERPs) may offer critical information about the neurophysiological mechanisms underlying the SMC deficits in decision making. In the present study, we focus on two ERP components called Feedback-related negativity (FRN) and the P3 during the feedback evaluation stage. On the one hand, the FRN component is a negative brain response that appears 260 ms after the presentation of the feedback and reaches its maximum amplitude in frontal-central scalp areas (Gehring and Willoughby, 2002). On decision-making tasks, FRN is modulated by the feedback outcome, where losses present a greater amplitude compared to wins (Cui et al., 2013; Tamburin et al., 2014). Therefore, FRN reflects an early feedback evaluation (i.e., good vs bad outcome) that modulates the reward system that guides the learning process. Moreover, FRN may be conceived as reward-related positivity, where positivity is greater for rewarded feedback than for non-rewarded feedback (Proudfit, 2015; Kallen et al., 2020; Threadgill et al., 2020; Bowyer et al., 2021; Burani et al., 2021). On the other hand, the P3 component is a positive brain response that emerges around 300–450 ms after feedback presentation and reaches its maximum amplitude in the parietal-central midline areas (Carlson et al., 2009; Cui et al., 2013; Tamburin et al., 2014). The P3 component is modulated by the magnitude and valence of the feedback outcome, with wins showing larger amplitudes than losses on a monetary gambling task (Hewig et al., 2006; Gu et al., 2010). Thus, the P3 component reflects later feedback evaluation processes in which the amplitude is generally considered a measure of memory workload. These ERP components would provide critical evidence about the influence of SMCs on decision-making processes.

It is important to note that, although SMCs have been especially investigated in older people (Ponds et al., 1997), recent evidence indicates that age may not be the cause of the appearance of SMCs (Rowell et al., 2015). Along these lines, previous research has shown that SMCs are also present in young adults (Derouesné et al., 1999; Mendes et al., 2008; Pearman, 2009; Molina-Rodríguez et al., 2018), and it has been suggested that SMCs are equally frequent in young and older adults, but qualitatively different (Ginó et al., 2010). Moreover, research has shown a decline in decision-making processes as people age (Beitz et al., 2014), and current evidence indicates age-related changes in the neurophysiological mechanisms underlying decision making. For instance, aging has been associated with a decline in prefrontal cortex functioning that causes a weakening of negative feedback processing mediated by dopamine modulation in the anterior cingulate cortex (Glimcher, 2011). In addition, older adults present less difference in the amplitude between losses and wins than young adults in the FRN component (Hämmerer et al., 2011), suggesting that older adults find it more difficult to learn the contingencies of the feedback evaluation. Additionally, studies have shown that the P3 amplitude was significantly reduced in older adults compared to young adults after negative feedback in the risky phase of the IGT (Di Rosa et al., 2017). These studies indicate that processes related to decision making may be different in young and older people. Thus, it is important to investigate whether these differences are also present in young and older people with SMCs, and whether young and older people with and without SMCs may differ on these physiological markers.

The aim of this study was to investigate whether young and older participants with and without SMCs differ on behavioral and neural correlates (i.e., FRN and P3) of decision making during the IGT. Regarding behavioral data, we hypothesized that the performance on the IGT would be worse for groups with SMCs than for groups without SMCs. Moreover, following Smart and Krawitz (2015), we expected that the performance of the SMC group, regardless of age, would be lower in the risky phase than in the ambiguity phase. Regarding the electrophysiological data, ERP components were analyzed in the feedback evaluation stage, and we expected to observe delayed latencies and decreased FRN and P3 amplitudes in the SMCs participants, especially for losses, which would reflect deficits in feedback processing.

## Materials and Methods

### Participants

A total of 160 participants were recruited for this study. After excluding participants with depressive symptomatology (with a score above 14 on the Beck Depression Inventory-II; Beck et al., 1961, 1996), the final sample included 136 participants, of whom 65 were young adults (32 men, 33 women) between 18 and 34 years old, with a mean age of 22.4 years (SD = 3.77), and 71 were older adults (37 men, 34 women) between 55 and 75 years old, with a mean age of 64.5 years (SD = 5.62). The young sample was composed of undergraduate students from different bachelor’s degrees at the University of Valencia. The older sample was recruited in university courses and seminars offered by the University of Valencia for people over 55 years old. Before being included in the study, participants completed a telephone interview to ensure that they met the following inclusion criteria: not smoking more than 10 cigarettes a day; no record of neurological, psychiatric, or psychological disorders; not taking any medication that affected the central nervous and endocrine system; no drug or alcohol abuse; not having undergone surgery with anesthesia in the past year; and not experiencing any stressful events in at least the past 6 months (i.e., death of a loved one). All participants were right-handed, measured by the Edinburgh Handedness Inventory (Oldfield, 1971). The young sample without SMCs was part of the study sample reported in Garrido-Chaves et al. (2020).

Young and older adults were divided according to the scores obtained on the Spanish version of the Memory Failures of Everyday questionnaire (MFE-30; Sunderland et al., 1984; Spanish adaptation by Lozoya-Delgado et al., 2012). The MFE-30 contains 30 items rated on a 5-point Likert scale (ranging from 0 = never or almost never, to 4 = always or almost always). This questionnaire is employed in research and clinical settings to measure subjective memory failures of everyday life (sample items are: “I forget where I have put something. I lose things at home” and “I have a word “on the tip of my tongue.” I know what I want to say but I can’t find the right expression”). The psychometric properties of this questionnaire showed that it is unifactorial, and that its Cronbach’s alpha is 0.93 (Pedrero-Pérez et al., 2011). Importantly, the use of cut points and categorical distinctions is commonly used in clinical practice and may be helpful to neuropsychologists. Lozoya-Delgado et al. (2012) showed that 21 was the mean score on this questionnaire in a sample of 900 Spanish participants. Based on this result and our previous study (Perez et al., 2021), participants with a score of 21 or higher on the MFE-30 were included in the SMCs group. Participants who scored below 21 were included in the group with no SMCs (noSMCs). Therefore, participants were divided into four groups: young adults with SMCs (young SMCs; *n* = 28; 11 men, 17 women); young adults without SMCs (young noSMCs; *n* = 37; 21 men, 16 women); older adults with SMCs (older SMCs; *n* = 32; 14 men, 18 women); and older adults without SMCs (older noSMCs; *n* = 39; 23 men, 16 women).

The SMC construct implies the absence of objective cognitive impairment (Steinberg et al., 2013). The sample of older adults performed the Mini-Mental State Examination (MMSE; Folstein et al., 1975), which is the most commonly used test to detect and screen for cognitive impairment and dementia (Folstein et al., 1975). No differences were observed between groups on the MMSE (SMCs: mean = 28.8, SD = 1.3; noSMCs: mean = 29.0, SD = 1.6) (*p* = 0.644). Moreover, to exclude participants with cognitive impairment, all the subjects participated in a neuropsychological assessment to evaluate EF and attention (Trail Making Test Part A and B, the Stroop test, and the Digit Span forward and backward), visuo-spatial and verbal short- and long-term memory (Rey–Osterrieth complex figure and Free and Cued Selective Reminding Test), and language (phonological and semantic fluency tests) (Reitan, 1958; Golden and Freshwater, 1978; Meyers and Meyers, 1995; Wechsler, 1997; Tombaugh et al., 1999). Importantly, according to the Spanish multicenter normative studies (NEURONORMA project; Peña-Casanova et al., 2009b,c; Tamayo et al., 2012; Casals-Coll et al., 2013; Rognoni et al., 2013), the results on all the neuropsychological tests were in the normal range for all the participants. These results confirm the absence of objective cognitive impairment in the participants in the SMC and noSMC groups. The results of these tests are not part of the research topic of the current study and will be published elsewhere; therefore, they are not reported here.

### Procedure

All participants signed an informed consent prior to the study. This study was approved by the ethical committee of the University of Valencia and met the Declaration of Helsinki criteria. Each participant completed two experimental sessions that were conducted in the Laboratory of Social Cognitive Neuroscience at the University of Valencia. The sessions were held on two consecutive days and lasted 2 h each. On the first day, the participants completed the neuropsychological evaluation used to exclude participants with cognitive impairment. On the second day, the participants performed the decision-making task, an adapted version of the original IGT for studying ERPs (Bechara et al., 1994). Before the IGT, participants performed a face emotion recognition task and a semantic go/no-go task (results will be published elsewhere). They were asked not to smoke cigarettes or drink alcohol or coffee at least 2 h before the two experimental sessions. Participants were randomly assigned to start the session at 10 a.m., 12 a.m., 3 p.m., or 5 p.m. The participants were seated in a comfortable chair at a distance of 70 cm from the monitor screen; the visual angle was ∼4.5°.

### Iowa Gambling Task

**FIGURE 1:**
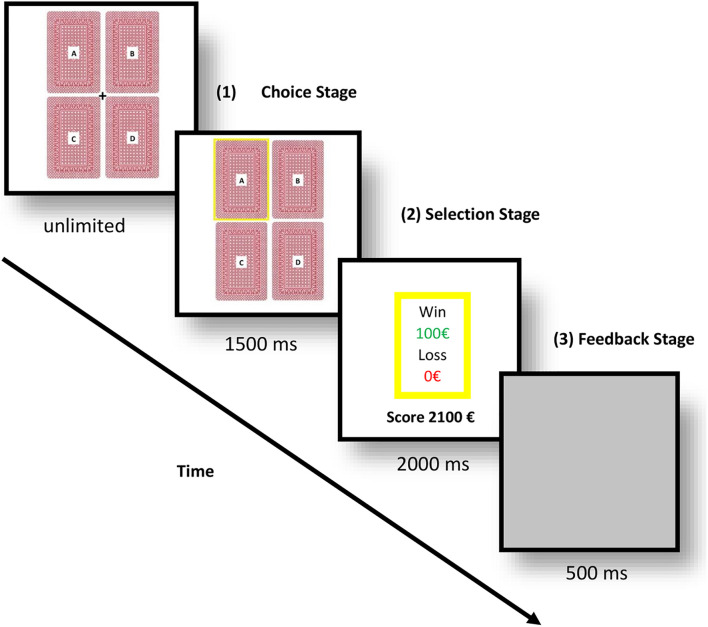
Experimental flow of the adapted version of the Iowa gambling task (IGT) for event-related potentials (ERPs).

We used an adapted version of the classical IGT for ERPs, see Figure 1 (Bechara et al., 1994). The participants were told that the task had no limit time, and that they could choose whatever deck they wanted, but each selection could lead to a cost or reward. All the participants read the same instructions, and they were told to earn as much money as possible. They started with 2000 € in virtual money, and they had to perform a decision-making task with four decks labeled A, B, C, or D. Decks A and B were the disadvantageous decks because they brought greater immediate wins, but greater losses in the long term. The other two decks (C and D) were the advantageous decks because they led to small immediate wins and smaller losses in the long term. The advantageous and disadvantageous decks were assigned in the same distribution and proportion as in the original IGT (Bechara et al., 1994). The participants were unaware of the contingencies of the decks or the distribution of the rewards before the experiment.

These four decks were presented on the screen in two rows in order to reduce eye movement. After participants had picked a deck, the selected deck was surrounded by a yellow frame. After 1,500 ms, they received positive or negative feedback, and the accumulated earned money appeared on the center of the screen for 2,000 ms, followed by a gray screen for 500 ms, which indicated the end of the trial. In order to complete the task, each participant had to pick one deck at a time during 150 trials; however, for the purposes of this study, only the first 100 trials were included in the analyses. Each deck contained a total of 40 cards, and when the cards in a deck ran out, an informative pop-up window appeared on the screen indicating that they had to choose a different deck. All the participants chose a deck by moving the cursor and then clicking on the left mouse button with their right forefinger.

For the behavioral data, we divided the 100 trials into five blocks of 20 trials each, as in previous studies using the classical IGT (Bechara et al., 1994). The IGT performance was measured with the IG index, which was analyzed by calculating the formula [(C + D) − (A + B)]. The IG index is the difference between the number of trials of the advantageous decks minus the number of trials of the disadvantageous decks in each of the five blocks of 20 trials for each participant. The IG index is considered a learning measure; therefore, scores above zero indicate better task performance, whereas scores below zero indicate worse task performance. Regarding ERP data, we also analyzed the first 100 trials, which were divided into two blocks of 50 trials each, where the first block (1–50 trials) corresponds to the ambiguity phase and the second block (51–100 trials) to the risky phase, as in the classical IGT.

### Electroencephalogram Recording and Analysis

Electroencephalogram (EEG) was recorded by means of 29 active electrodes using a Brain Vision amplifier system (BrainProducts, Germany). Twenty-two of these electrodes (Fpz, Fp1, Fp2, Fz, F7, F3, F4, F8, FCz, Cz, C3, C4, T7, T8, Pz, P3, P4, P7, P8, Oz, O1, and O2) were placed on an elastic cap (Easycap, Falk Minow, Munich, Germany), according to the International 10–20 system. The seven remaining electrodes were applied on the mastoids (M1, M2), on the external canthus of each eye (HEOG+, HEOG−), and supra-and infraorbitally to the right eye (VEOG+, VEOG−), and the ground electrode was placed on AFz. The average of the two mastoids was computed offline as the reference for all EEG channels. The EEG signal was filtered through a 0.01–100 Hz analog band-pass filter and registered with a sampling rate of 500 Hz. Signal preprocessing was performed offline using a standard pipeline for ERP data with the BrainVision Analyzer (BrainProducts, Germany). First, all signal was visually inspected to identify defective electrodes or artifacts, which were excluded from the averaging. Impedances were maintained below 5 kΩ. In order to remove blinks from analyses, we employed offline the Gratton et al. (1983).

The EEG signal was passed through a 0.1–30 Hz bandpass filter. One-second epochs were speared for the feedback stage, consisting of 200 ms before feedback presentation (corrected to baseline) and 800 ms after it. Epochs beyond ±50 μV were automatically rejected. The epochs were analyzed only in the feedback evaluation stage. We analyzed the amplitude and latency of the FRN and P3 for each type of feedback (i.e., losses and wins). ERP data were obtained by averaging the epochs. The FRN amplitude was calculated in the time window [230–350] ms after the onset of the feedback in the Fz and FCz channels because the FRN component peaks at the fronto-central midline sites (Gehring and Willoughby, 2002; Cui et al., 2013; Tamburin et al., 2014). The P3 amplitude was calculated in the time window [300–450] ms after feedback presentation in the Pz channel because it is largest on the scalp over parietal-central sites (Carlson et al., 2009; Cui et al., 2013; Tamburin et al., 2014). Mean amplitudes were calculated using the Peak Detection Method through semiautomatic detection. Each participant was visually inspected to mark the global maximum amplitude in both ERP components at the electrodes of interest.

### Statistical Analyses

Differences between the SMCs and noSMCs groups and between older and young people on the MFE-30 were examined using two independent *t*-tests. Differences in sex distribution were investigated by means of the Chi square test (χ^2^). For the IGT performance, the IG scores on the 100 trials were investigated using a rm-ANOVA with Block (1, 2, 3, 4, and 5) as the within-subject factor and Group (SMCs, noSMCs) and Age (Young, Older) as the between-subject factors. The Greenhouse- Geisser correction was used for effects of Block. ERP data were analyzed using parameters of amplitude and latency. For FRN, a rm-ANOVA was conducted, with Block (1 and 2), Feedback (wins and losses), and Electrode (Fz and FCz) as within-subject factors, and Group (SMCs, noSMCs) and Age (Young, Older) as between-subject factors. For the P3 component, a rm-ANOVA was performed, with Block (1 and 2) and Feedback (wins and losses) as within-subject factors, and Group (SMCs, noSMCs) and Age (Young, Older) as between-subject factors. *Post hoc* comparisons were employed using Bonferroni’s correction when rm-ANOVAs revealed a significant effect. Partial eta square (η^2^p) is provided as indicator of effect size. All statistical analyses were performed using IBM SPSS version 25. A significance threshold of *p* < 0.05 was used for all tests.

## Results

### Description of the Study Sample

Demographic data are reported in Table 1. As expected, MFE-30 scores were higher in the SMCs group than in the noSMCs group. No significant differences in MFE-30 between young and older participants were found. No Group (SMCs vs noSMCs) differences in age or the proportion of men and women were found.

**TABLE 1:** Demographic information.

**Mean (SD)**	**Young SMCs**	**Older SMCs**	**Young noSMCs**	**Older noSMCs**	**Differences between Groups (SMCs vs noSMCs)**
*N*	28	32	37	39	
Sex (females)	17	18	16	16	χ^2^ = 3.533, *p* = 0.060
Age (years)	21.3 (3.3)	63.7 (5.6)	23.3 (3.9)	65.2 (5.6)	*t*_(__134__)_ = 0.232, *p* = 0.817
MFE-30	34.1 (11.2)	38.3 (12.5)	10.5 (5.3)	12.15 (5.9)	***t*_(__134__)_ = 16.054, *p* < 0.001**

*Means ± standard deviations (SD) for the demographic information by group.*

*SMCs, group with subjective memory complaints; noSMCs, group without subjective memory complaints.*

*In bold significant differences (*p* ≤ 0.050).*

### Iowa Gambling Task Performance

To investigate differences in behavioral performance on the IGT (young SMCs vs older SMCs vs young noSMCs vs older noSMCs), we carried out a rm-ANOVA with Block (1, 2, 3, 4, and 5) as the within-subject factor, Group (SMCs, noSMCs) and Age (young, older) as the between-subject factors, and the IG scores on the 100 trials as the dependent variable.

**FIGURE 2:**
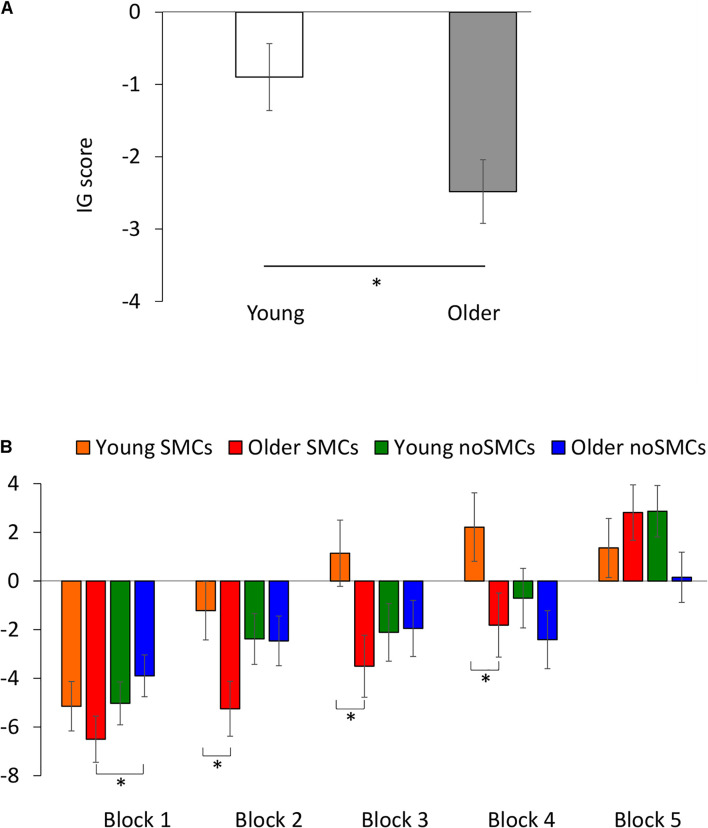
Behavioral performance on the IGT. **(A)** IG index for young and older adults after 100 trials. **(B)** IG index for the interaction between Block, subjective memory complaints (SMCs), and Age. Means (±SEM) are shown. *p < 0.05.

The rm-ANOVA for the IG score on the 100 trials did not reveal a significant effect of Group [*F*(1,132) = 0.100, *p* = 0.752, η_*p*_2 = 0.001]. However, a significant effect of Block [*F*(3.671,484.580) = 23.531, *p* < 0.001, η_*p*_2 = 0.151] was observed. In general, participants scored better in the fifth block than in the first (*p* = 0.003), second (*p* < 0.001), third (*p* < 0.001), and fourth (*p* < 0.001) blocks, and they scored worse between the first and second (*p* = 0.001), third (*p* < 0.001), and fourth (*p* < 0.001) blocks. No differences were found between the second and third (*p* = 0.600) and fourth (*p* = 0.055) blocks, or between the third and fourth (*p* > 0.999) blocks. There was a significant main effect of Age [*F*(1,132) = 6.144, *p* = 0.014, η_*p*_2 = 0.044], with young adults scoring better than older adults (see Figure 2A). Importantly, a significant interaction between Block × Group × Age was observed [*F*(3.671,484.580) = 2.783, *p* = 0.030, η_*p*_2 = 0.021], showing that the young SMCs group showed a better IG score than the older SMC group in the second (*p* = 0.016), third (*p* = 0.014), and fourth (*p* = 0.039) blocks, but not in the first (*p* = 0.331) and fifth blocks (*p* = 0.384; see Figure 2B). Moreover, the older noSMCs group showed a higher IG score than the older SMCs group only in the first block (*p* = 0.044). None of the other interactions were statistically significant (all *p* > 0.143).

### Event-Related Potential Results

#### Feedback-Related Negativity Results

To investigate group and age differences in FRN (young SMCs vs older SMCs vs young noSMCs vs older noSMCs), we carried out rm-ANOVA with Block (1 and 2), Feedback (wins and losses), and Electrode (Fz and FCz) as within-subject factors, and Group (SMCs, noSMCs) and Age (young, older) as between-subject factors. The parameters of amplitude and latency were used as dependent variables.

**FIGURE 3:**
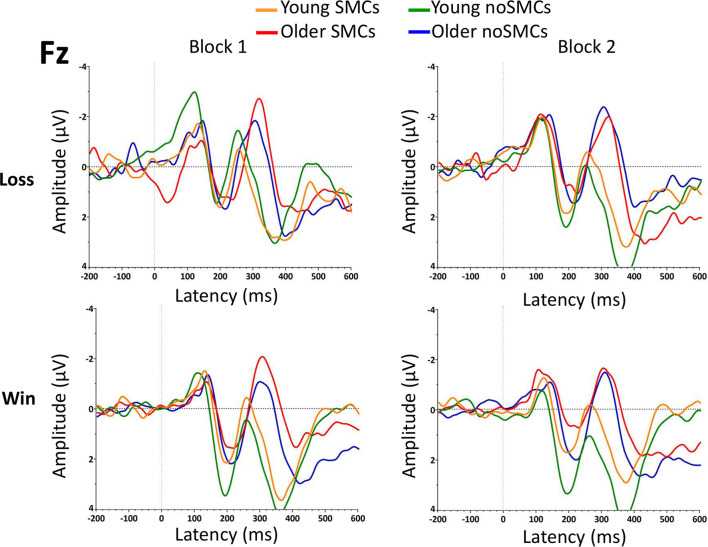
Grand averages of the feedback-related negativity (FRN) component at the Fz electrode for the two blocks.

**FIGURE 4:**
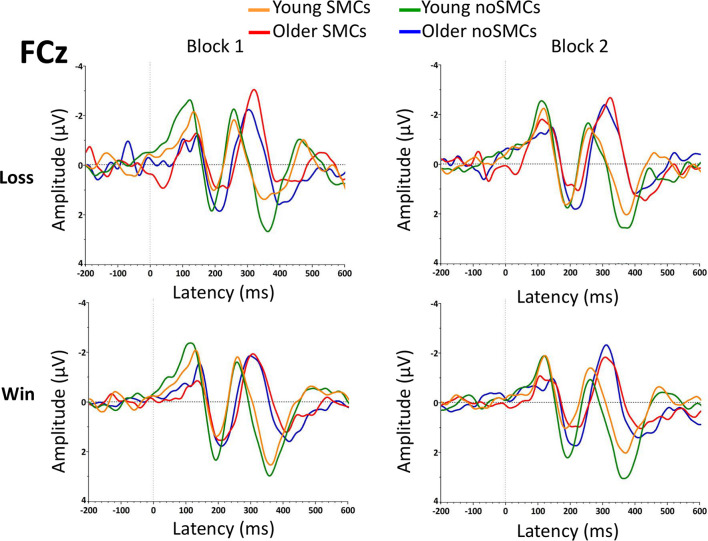
Grand averages of the FRN component at the FCz electrode for the two blocks.

The results of the rm-ANOVA for amplitudes and latencies of the FRN are presented in Tables 2, 3. In addition, grand averages of the waveforms are presented in Figure 3 (Fz electrode) and Figure 4 (FCz electrode).

**TABLE 2:** Event-related potential (ERP) results.

	**Young SMCs**		**Older SMCs**		**Young noSMCs**		**Older noSMCs**	
**Block**	**1**	**2**	**1**	**2**	**1**	**2**	**1**	**2**
***FRN* (*Fz*)**								
Losses (μV)	−1.7 (0.6)	−2.2 (0.7)	−3.8 (0.7)	−3.2 (0.8)	−2.7 (0.7)	−1.4 (0.7)	−3.3 (0.6)	−4.1 (0.7)
Wins (μV)	−1.5 (0.5)	−1.3 (0.5)	−3.0 (0.6)	−2.8 (0.6)	−0.6 (0.6)	−0.3 (0.5)	−2.0 (0.5)	−2.2 (0.5)
Losses (ms)	267 (5)	266 (6)	318 (4)	300 (5)	261 (5)	262 (5)	299 (5)	311 (5)
Wins (ms)	268 (5)	264 (5)	312 (4)	313 (5)	260 (4)	265 (4)	306 (5)	307 (4)
***FRN* (*FCz*)**								
Losses (μV)	−3.0 (0.5)	−3.2 (0.4)	−4.3 (0.3)	−3.9 (0.5)	−3.7 (0.5)	−3.2 (0.4)	−3.7 (0.3)	−4.1 (0.4)
Wins (μV)	−2.6 (0.3)	−2.3 (0.3)	−3.0 (0.4)	−3.0 (0.3)	−2.4 (0.4)	−2.2 (0.3)	−3.1 (0.3)	−3.1 (0.3)
Losses (ms)	269 (5)	267 (6)	320 (5)	302 (6)	263 (5)	261 (4)	299 (5)	310 (5)
Wins (ms)	267 (5)	264 (5)	311 (4)	313 (5)	259 (4)	265 (4)	303 (5)	311 (4)
***P3* (*Pz*)**								
Losses (μV)	9.7 (0.7)	10.3 (1.0)	7.8 (0.7)	7.9 (0.8)	8.8 (0.9)	9.0 (1.0)	8.2 (0.7)	8.3 (1.4)
Wins (μV)	10.6 (0.8)	10.1 (0.7)	7.0 (0.6)	7.0 (0.6)	10.1 (0.8)	8.5 (0.8)	7.4 (0.6)	7.7 (0.6)
Losses (ms)	365 (6)	367 (7)	401 (9)	395 (7)	368 (10)	373 (5)	388 (7)	387 (7)
Wins (ms)	362 (5)	365 (5)	388 (9)	396 (8)	366 (7)	36 5 (7)	384 (6)	396 (7)

*Mean ± SEM (in brackets) of amplitudes (μV) and latencies (ms) of the FRN and P3 components divided by group and block.*

*SMCs, group with subjective memory complaints; noSMCs, group without subjective memory complaints.*

**TABLE 3:** ERP results.

**rm-ANOVAs**	**FRN (amplitude)**	**FRN (latency)**	**P3 (amplitude)**	**P3 (latency)**
Feedback	***F* = 42.98, *p* < 0.01, η_*p*_^2^ = 0.246**	*F* = 0.29, *p* = 0.59, η_*p*_^2^ = 0.002	*F* = 0.62, *p* = 0.43, η_*p*_^2^ = 0.005	*F* = 0.96, *p* = 0.33, η_*p*_^2^ = 0.007
Block	*F* = 0.47, *p* = 0.49, η_*p*_^2^ = 0.004	*F <* 0.01, *p* = 0.97, η_*p*_^2^< 0.001	*F* = 0.11, *p* = 0.74, η_*p*_^2^ = 0.001	*F* = 1.00, *p* = 0.32, η_*p*_^2^ = 0.008
Electrode	***F* = 28.15, *p* < 0.01, η_*p*_^2^ = 0.176**	*F* = 0.71, *p* = 0.40, η_*p*_^2^ = 0.005	—	—
Age	***F* = 9.37, *p < 0*.05, η_*p*_^2^ = 0.066**	***F* = 162.81, *p* < 0.01, η_*p*_^2^ = 0.552**	***F* = 8.26, *p < 0*.01, η_*p*_^2^ = 0.059**	***F* = 24.26, *p* < 0.01, η_*p*_^2^ = 0.155**
Group	*F* = 0.20, *p* = 0.65, η_*p*_^2^ = 0.002	*F* = 2.13, *p* = 0.15, η_*p*_^2^ = 0.016	*F* = 1.24, *p* = 0.27, η_*p*_^2^ = 0.009	*F* = 0.83, *p* = 0.36, η_*p*_^2^ = 0.364
Group × Age	*F* < 0.01, *p* = 0.97, η_*p*_^2^< 0.001	*F* = 0.02, *p* = 0.89, η_*p*_^2^< 0.001	*F* = 0.23, *p* = 0.64, η_*p*_^2^ = 0.002	*F* = 0.09, *p* = 0.77, η_*p*_^2^ = 0.001
Feedback × Block	*F* = 0.01, *p* = 0.90, η_*p*_^2^< 0.001	*F* = 1.58, *p* = 0.21, η_*p*_^2^ = 0.012	*F* = 3.05, *p* = 0.08, η_*p*_^2^ = 0.023	*F* = 1.31, *p* = 0.25, η_*p*_^2^ = 0.010
Feedback × Electrode	*F* = 0.70, *p* = 0.41, η_*p*_^2^ = 0.005	*F* = 1.91, *p* = 0.17, η_*p*_^2^ = 0.014	—	—
Feedback × Age	*F* = 0.07, *p* = 0.79, η_*p*_^2^ = 0.001	*F* = 0.91, *p* = 0.34, η_*p*_^2^ = 0.007	***F* = 4.19, *p < 0*.05, η_*p*_^2^ = 0.031**	*F* = 0.07, *p* = 0.80, η_*p*_^2^ = 0.001
Feedback × Group	*F* = 3.52, *p* = 0.06, η_*p*_^2^ = 0.026	*F* = 0.09, *p* = 0.77, η_*p*_^2^ = 0.001	*F* = 0.01, *p* = 0.91, η_*p*_^2^ < 0.001	*F* = 0.77, *p* = 0.38, η_*p*_^2^ = 0.006
Block × Electrode	*F* = 0.01, *p* = 0.92, η_*p*_^2^< 0.001	*F* = 0.58, *p* = 0.45, η_*p*_^2^ = 0.004	—	—
Block × Age	*F* = 0.69, *p* = 0.41, η_*p*_^2^ = 0.005	*F* = 0.01, *p* = 0.94, η_*p*_^2^ ≤ 0.001	*F* = 0.45, *p* = 0.51, η_*p*_^2^ = 0.003	*F* = 0.06, *p* = 0.81, η_*p*_^2^< 0.001
Block × Group	*F<*0.01, *p* = 0.97, η_*p*_^2^< 0.001	***F* = 6.20, *p < 0*.05, η_*p*_^2^ = 0.045**	*F* = 0.53, *p* = 0.47, η_*p*_^2^ = 0.004	*F* = 0.26, *p* = 0.61, η_*p*_^2^ = 0.002
Electrode × Age	***F* = 6.16, *p <* 0.05, η_*p*_^2^ = 0.045**	*F<*0.01, *p* = 0.98, η_*p*_^2^ < 0.001	—	—
Electrode × Group	*F* = 1.09, *p* = 0.30, η_*p*_^2^ = 0.008	*F* = 0.16, *p* = 0.69, η_*p*_^2^ = 0.001	—	—
Feedback × Block × Electrode	*F* = 0.01, *p* = 0.93, η_*p*_^2^< 0.001	*F* = 3.23, *p* = 0.08, η_*p*_^2^ = 0.024	—	—
Feedback × Block × Age	*F<*0.01, *p* = 0.99, η_*p*_^2^< 0.001	*F* = 0.27, *p* = 0.60, η_*p*_^2^ = 0.002	*F* = 3.20, *p* = 0.08, η_*p*_^2^ = 0.024	*F* = 2.37, *p* = 0.13, η_*p*_^2^ = 0.018
Feedback × Block × Group	*F* = 0.04, *p* = 0.85, η_*p*_^2^< 0.001	*F* = 1.89, *p* = 0.17, η_*p*_^2^ = 0.014	*F* = 0.36, *p* = 0.55, η_*p*_^2^ = 0.003	*F* = 0.05, *p* = 0.82, η_*p*_^2^< 0.001
Feedback × Electrode × Age	*F* = 0.02, *p* = 0.89, η_*p*_^2^< 0.001	*F* = 0.02, *p* = 0.89, η_*p*_^2^ < 0.001	—	—
Feedback × Electrode × Group	***F* = 6.31, *p < 0*.05, η_*p*_^2^ = 0.046**	*F* = 1.50, *p* = 0.22, η_*p*_^2^ = 0.011	—	—
Feedback × Age × Group	*F* = 0.51, *p* = 0.48, η_*p*_^2^ = 0.004	*F* = 0.17, *p* = 0.68, η_*p*_^2^ = 0.001	*F* = 0.01, *p* = 0.91, η_*p*_^2^< 0.001	*F* = 0.26, *p* = 0.61, η_*p*_^2^ = 0.002
Block × Electrode × Age	*F* = 0.31, *p* < 0.58, η_*p*_^2^ = 0.002	*F* = 2.15, *p* = 0.15, *η*_*p*_*^2^* = 0.016	—	—
Block × Electrode × Group	*F* = 0.05, *p* = 0.82, η_*p*_^2^< 0.001	*F* = 0.03, *p* = 0.86, η_*p*_^2^< 0.001	**—**	**—**
Block × Age × Group	*F* = 3.60, *p* = 0.06, η_*p*_^2^ = 0.027	*F* = 1.88, *p* = 0.17, η_*p*_^2^ = 0.014	*F* = 0.20, *p* = 0.66, η_*p*_^2^ = 0.002	*F* = 0.09, *p* = 0.77, η_*p*_^2^ = 0.001
Electrode × Age × Group	*F* = 0.24, *p* = 0.62, η_*p*_^2^ = 0.002	*F* = 0.20, *p* = 0.65, η_*p*_^2^ = 0.002	—	—
Feedback × Block × Electrode × Age	*F* = 0.72, *p* = 0.40, η_*p*_^2^ = 0.005	*F* = 0.24, *p* = 0.62, η_*p*_^2^ = 0.002	—	—
Feedback × Block × Electrode × Group	*F* = 0.33, *p* = 0.57, η_*p*_^2^ = 0.003	*F* = 0.77, *p* = 0.38, η_*p*_^2^ = 0.006	—	—
Feedback × Block × Age × Group	*F* = 2.39, *p* = 0.12, η_*p*_^2^ = 0.018	***F* = 6.33, *p < 0*.05, η_*p*_^2^ = 0.046**	*F* = 0.40, *p* = 0.80, η_*p*_^2^ = 0.001	*F* = 0.14, *p* = 0.71, η_*p*_^2^ = 0.001
Feedback × Electrode × Age × Group	*F* = 0.83, *p* = 0.36, η_*p*_^2^ = 0.006	*F* = 0.78, *p* = 0.38, η_*p*_^2^ = 0.006	—	—
Block × Electrode × Age × Group	*F* = 2.47, *p* = 0.12, η_*p*_^2^ = 0.018	*F* = 1.01, *p* = 0.31, η_*p*_^2^ = 0.008	—	—
Feedback × Block × Electrode × Age × Group	*F* = 1.02, *p* = 0.31, η_*p*_^2^ = 0.008	*F* = 0.36, *p* = 0.55, η_*p*_^2^ = 0.003	—	—

*Note:η_*p*_^2^ = partial eta square; in bold significant difference (*p* < 0.050). F’s degrees of freedom = 1,132.*

##### Amplitude

The rm-ANOVA for the FRN amplitude showed a significant main effect of Feedback (*p* < 0.001) and Age (*p* = 0.003). Losses evoked larger amplitudes than wins, and a larger amplitude was observed for older adults than for young adults.

The Electrode factor was statistically significant, with FCz showing a larger amplitude than Fz (*p* < 0.001). The following interactions were also statistically significant: Electrode × Age (*p* = 0.014), and Feedback × Electrode × Group (*p* = 0.013). Although we observed an interaction between feedback, electrode, and group, the *post hoc* analyses revealed no significant differences between the SMCs and noSMCs groups (all *p* > 0.119). Overall, these interactions indicate a larger amplitude at the FCz than at the Fz electrode in both young (*p* < 0.001) and older (*p* < 0.043) adults. As observed for the main effect of Age, a larger amplitude was observed in older people compared to young adults at both the Fz (*p* < 0.002) and FCz (*p* < 0.019) electrodes. Moreover, the effect of Feedback was statistically significant for both electrodes in the noSMCs group (*p* < 0.001). In the SMCs group, the difference was statistically significant at the FCz electrode (*p* < 0.001). However, this difference did not reach statistical significance at the Fz electrode in the SMCs group (*p* = 0.078). Importantly, the *post hoc* analyses indicate that, at each electrode, the amplitudes for wins and losses were similar in both groups (Fz wins noSMCs vs Fz wins SMCs: *p* = 0.089; Fz losses noSMCs vs Fz losses SMCs *p* = 0.780; FCz wins noSMCs vs FCz wins SMCs: *p* = 0.877, FCz losses noSMCs vs losses SMCs: *p* = 0.834.

Importantly, the Group factor (SMCs vs noSMCs) and the interactions with the other factors were not statistically significant (all *p* > 0.060).

##### Latency

The rm-ANOVA for the FRN latency showed a significant main effect of Age (*p* < 0.001). Older adults presented a more delayed latency than young adults. In addition, the following interactions were statistically significant: Block × Group (*p* < 0.014) and Block × Feedback × Age × Group (*p* < 0.013). Regarding the Block × Group interaction, the results indicate that participants with SMCs showed a delayed latency when compared with participants with noSMCs in the first block (*p* = 0.009), but not in the second block (*p* = 0.934), and no differences were found between the first and second blocks in the SMCs (*p* = 0.094) and noSMCs (*p* = 0.067) groups. Additionally, the Block × Feedback × Age × Group interaction showed that older adults with SMCs evoked a delayed latency compared to older adults from the noSMCs group only for losses in the first block (*p* = 0.002). Furthermore, older people in the noSMCs group (*p* = 0.042) showed a longer latency for losses in the second block than in the first block. Older people with SMCs (*p* = 0.004) showed a longer latency for losses in the first block than in the second block. Finally, older people with SMCs showed a longer latency for wins than for losses only in the second block (*p* = 0.010). None of the other *post hoc* analyses were statistically significant (all *p* > 0.075).

#### P3 Results

To investigate group and age differences in P3 (young SMCs vs older SMCs vs young noSMCs vs older noSMCs), we carried out rm-ANOVA with Block (1 and 2) and Feedback (wins and losses) as within-subject factors and Group (SMCs, noSMCs) and Age (young, older) as between-subject factors. The parameters of amplitude and latency were used as dependent variables.

**FIGURE 5:**
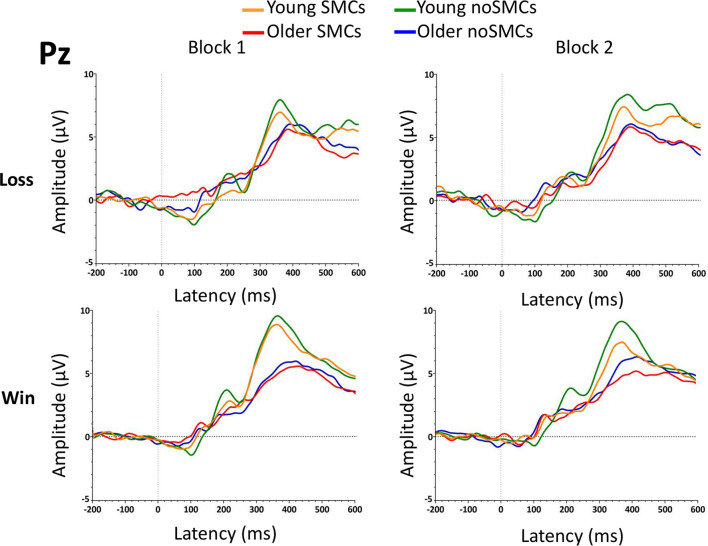
Grand averages of the P3 component at the Pz electrode for the two blocks.

The results of the rm-ANOVA for the P3 amplitudes and latencies are presented in Tables 2, 3. In addition, the grand average of the waveform is presented in Figure 5.

##### Amplitude

The rm-ANOVA of the P3 amplitude in the Pz electrode showed a significant main effect of Age (*p* = 0.005), with young adults evoking a significantly larger P3 amplitude than older adults. Moreover, a significant interaction between Feedback × Age was found. Older adults presented a larger amplitude for losses than wins (*p* = 0.042), but no differences were found between losses and wins in the young adults (*p* = 0.385). Older adults evoked a smaller P3 amplitude than young adults for wins (*p* < 0.001), but not for losses (*p* = 0.097).

The Group factor (SMCs, noSMCs) and the interactions with other factors were not statistically significant (*p* > 0.267).

##### Latency

Regarding the P3 latency in the Pz electrode, only a significant effect of Age (*p* < 0.001) was observed, showing that older people evoked a delayed P3 latency compared to young adults.

Finally, the Group factor (SMCs, noSMCs) and the interactions with other factors were not statistically significant (*p* > 0.364).

## Discussion

The main objective of this study was to investigate the behavioral response and neural correlates of the decision-making process during the IGT in young and older adults with and without SMCs. Overall, we observed that the decision-making process in older people was affected by SMCs at a behavioral and electrophysiological level. Regarding the behavioral performance, older people without SMCs (older noSMCs) presented a better IG score than older people with SMCs (older SMCs) in the ambiguity phase and in the first part of the risky phase. Moreover, young people with SMCs (young SMCs) presented a higher IG index in the ambiguity and risky phases than older people with SMCs (older SMCs) did. Young adults showed a higher IG index and outperformed older adults. Regarding the electrophysiological results, the FRN latency was longer for losses in older people with SMCs than in older people without SMCs in the first block. No direct group differences were observed in the amplitude of the FRN component, and the amplitude was larger after losses than after wins (although this difference did not reach statistical significance in the SMCs group for the Fz electrode, *p* = 0.078). Furthermore, there were no Group (SMCs, noSMCs) differences in the amplitude and latency of the P3 component. We observed that older adults showed a larger amplitude and a more delayed latency than young adults in the FRN and P3 components.

To the best of our knowledge, this is the first ERP study to evaluate the decision-making process in young and older participants with SMCs. We observed differences in the IGT performance and in the FRN component between older people with and without SMCs in the ambiguity phase, and in behavioral performance during the transition from the ambiguity phase to the risky phase, which is when participants begin to explore the benefits of the decks. Our results indicate worse performance on the first 20 trials and longer latency in the FRN component on the first 50 trials in older people with SMCs than in older people without SMCs. These results suggest that older adults with SMCs, compared to older people without SMCs, have a specific difficulty in processing negative information during the ambiguity phase of the decision-making process, leading to worse IGT performance. Thus, older people with SMCs may have difficulties in processing negative outcomes when making decisions in ambiguous conditions because they need more time (i.e., delayed latency for losses) to process and avoid negative experiences and emotions. This deficit may have possible consequences for working memory updating, leading to worse decision-making performance (Depping and Freund, 2011). The results are in line with a previous study showing reduced future-oriented choices in older individuals with subjective cognitive decline (Hu et al., 2017). Moreover, Smart and Krawitz (2015) observed that older people with subjective cognitive decline presented difficulties in the risky phase. Although in our results we did not find differences between older people with and without SMCs in the later part of the risky phase (i.e., last 20 trials), we observed that young adults with SMCs (young SMCs) scored better in the ambiguity and risky phases than older people with SMCs (older SMCs). This could be explained by the fact that SMCs comprise a specific aspect of the memory function itself, and, therefore, previous experiences may be harder to recall for older people with SMCs than for young adults with SMCs. Overall, the current evidence indicates that the decision-making process may be affected in older people with SMCs, and that the deficits may depend on the characteristics of the task.

In contrast to the results in the FRN, we did not observe differences in amplitude and latency in P3, an ERP component that has been related to a later feedback evaluation (Wu and Zhou, 2009). The absence of SMCs differences in P3 during the IGT may be due to the fact that this decision-making task, despite being part of the EF, does not have a high attention requirement, compared to other EF tasks that have shown an effect of SMCs on neural correlates (Gironell et al., 2005; Smart et al., 2014; Cespón et al., 2018). Hu et al. (2017), using a future-oriented decision-making task, observed that future imagination increased future-oriented choices, and that it was associated with increased activation in the medial frontal polar cortex, right insular cortex, and anterior cingulate cortex in controls, but not in patients with SMCs. Thus, these results suggest that the main differences in decision-making processes between individuals with and without SMCs are due to functional differences in frontolimbic structures, associated with the FRN component, but not to parietal regions, associated with the P3 component. Our results further suggest that SMCs would be related to deficits in the early feedback process (i.e., reflected in FRN), but not in the later feedback process (i.e., reflected in P3). Future research is also needed to investigate the ERP correlates in other phases (e.g., selection phase) of the decision-making process.

In addition to the differences between the SMCs groups, we also observed an effect of age on behavioral and electrophysiological measures. In particular, older adults tended to choose more disadvantageous decks, as reflected in lower scores on the IG index compared to young participants. Thus, at the end of the IGT, older adults won less money than young adults. As expected, IGT performance declines with aging. These results are in line with previous studies showing IGT performance deficits and loss frequency bias in older adults (Beitz et al., 2014; Di Rosa et al., 2017). These differences between young and older adults could be explained by the fact that older adults employ a different strategy to compensate for their memory and EF decrease due to aging, thus affecting the decision-making process. This idea is supported by the electrophysiological results showing that losses evoked larger amplitudes than wins at the FRN [as observed in previous studies (Bianchin and Angrilli, 2011; Cui et al., 2013; Azcárraga-Guirola et al., 2016)]. Moreover, we observed higher FRN and lower P3 amplitudes, as well as greater FRN and P3 latencies, in older participants compared to young participants. These results may suggest that older adults increase their effort to recall past deck contingencies affecting the reward learning, which influences picking the advantageous decks on the IGT. Contrary to what was previously observed in the literature, older adults showed higher P3 amplitudes to losses than to wins (Hewig et al., 2006; Gu et al., 2010), supporting the notion of a decrease in the sensitivity of older adults to the type of feedback outcome (West et al., 2014). Finally, in line with the decline in processing speed hypothesis in older people (Salthouse, 1996), older adults presented a more delayed P3 latency than young adults, reflecting a slower feedback evaluation process (Porcaro et al., 2019).

In interpreting the results of this study, it is important to note that young participants were recruited from university students, and the sample of older people was recruited in university courses and seminars offered by a university. Thus, the high educational level of our sample should be considered when interpreting the results, given that it might affect the generalizability of our findings. Future studies may benefit from investigating the effect of SMCs on decision making in community samples.

## Conclusion

In conclusion, we observed that older people with SMCs present deficits in the decision-making process. These deficits are observed at the behavioral level, but also in neural mechanisms of early feedback processing of negative outcomes. Our results highlight the fact that SMCs and aging are critical factors in understanding the deficits in decision-making processes.

## Data Availability Statement

The raw data supporting the conclusion of this article will be made available by the authors, without undue reservation.

## Ethics Statement

This study was reviewed and approved by Ethics committee from the University of Valencia. The participants provided their written informed consent to participate in this study.

## Author Contributions

RG-C, MMP, and AS: study concept and design. RG-C, VP, MP-A, IC-S: acquisition of data. RG-C, MMP, MP-A, and TP: analysis and interpretation of the data. RG-C: drafting the manuscript. MMP, VH, and AS: revising the manuscript. MMP, VH, and AS: study supervision. All authors contributed to the article and approved the submitted version.

## Conflict of Interest

The authors declare that the research was conducted in the absence of any commercial or financial relationships that could be construed as a potential conflict of interest.

## Publisher’s Note

All claims expressed in this article are solely those of the authors and do not necessarily represent those of their affiliated organizations, or those of the publisher, the editors and the reviewers. Any product that may be evaluated in this article, or claim that may be made by its manufacturer, is not guaranteed or endorsed by the publisher.
